# Development and characterization of 3D spinal cord organoids to advance the study of amyotrophic lateral sclerosis

**DOI:** 10.1186/s43556-026-00453-0

**Published:** 2026-04-24

**Authors:** Matteo Bordoni, Eveljn Scarian, Letizia Messa, Maria Garofalo, Emanuela Jacchetti, Manuela Teresa Raimondi, Luca Diamanti, Stella Gagliardi, Stephana Carelli, Cristina Cereda, Orietta Pansarasa

**Affiliations:** 1 Cellular Models and Neuroepigenetics Unit, IRCCS Mondino Foundation, 27100 Pavia, Italy; 2 Department of Electronics, Information and Bioengineering (DEIB), Politecnico Di Milano, 20133 Milan, Italy; 3 Center of Functional Genomics and Rare Diseases, Department of Pediatrics, Buzzi Children’s Hospital, 20154 Milan, Italy; 4 Molecular Biology and Transcriptomics Unit, IRCCS Mondino Foundation, 27100 Pavia, Italy; 5 Department of Chemistry, Materials and Chemical Engineering “Giulio Natta”, Politecnico Di Milano, 20133 Milan, Italy; 6 Neuroncology Unit, IRCCS Mondino Foundation, 27100 Pavia, Italy; 7 Department of Biomedical and Clinical Sciences, Pediatric Clinical Research Center “Romeo Ed Enrica Invernizzi”, University of Milano, 20157 Milan, Italy

Dear Editor,

Organoid technology has emerged as a powerful tool to study neurodegenerative diseases (NDs) [1]. Human induced pluripotent stem cell (iPSC)-derived brain organoids self-organize into neural structures that recapitulate key aspects of early human brain development [1]. Amyotrophic lateral sclerosis (ALS) is a fatal ND characterized by progressive degeneration of upper and lower motor neurons (MNs) in the brain and spinal cord [2]. Although multiple molecular mechanisms contribute to ALS pathogenesis, increasing evidence highlights a critical role for non-neuronal cells [2]. To investigate these complex cellular interactions, biologically relevant human models are required. In this context, spinal cord organoids (SCOs) represent a promising approach. While previous studies have generated organoids from ALS patients carrying disease-associated mutations [3], here we provide a proof-of-concept for the generation of SCOs from sporadic ALS (sALS) subjects. This model represents an entry point to explore alterations in the extracellular microenvironment in sALS and may be further developed to study disease progression and support drug screening.

First, SCOs size was evaluated by light microscopy (Fig. 1a). During the early stages of SCOs differentiation, no differences were observed between sALS and healthy control (CTRL) SCOs. In contrast, at DIV28, sALS SCOs displayed a significantly reduced size compared with CTRL. Consistently, Nissl staining at DIV28 revealed disrupted morphology in sALS SCOs relative to CTRL (Fig. 1a), suggesting an earlier degenerative process. To investigate the ability of SCOs to develop and give rise to mature MNs, DIV28 SCOs were stained for the MN-specific markers HB9 and ISL1. A statistically significant decrease in HB9 expression was observed in sALS SCOs (54.4%, ****p < 0.0001) compared with CTRL SCOs (Fig. 1b), whereas no differences were detected in ISL1 expression. Neuronal and glial populations were further analyzed by staining for TUBB3, a neuron-specific marker, and GFAP, a glial marker. No statistically significant differences were detected in TUBB3 or GFAP overall expression levels. However, sALS SCOs displayed a markedly increased thickness of the GFAP⁺ cell layer (295.6%, ****p < 0.0001) compared with CTRL SCOs (Fig. 1b). We next evaluated Nestin, a marker of neural progenitor cells, and detected higher expression in sALS SCOs (131.1%, *p = 0.0133) compared with CTRL SCOs (Fig. 1b). The reduced size and altered morphology of sALS SCOs likely reflect neurodegenerative processes consistent with sALS hallmarks. These changes are accompanied by increased GFAP⁺ and Nestin⁺ cell populations and reduced HB9 expression, suggesting impaired neuronal maturation and a shift toward glial activation.

**Fig. 1:**
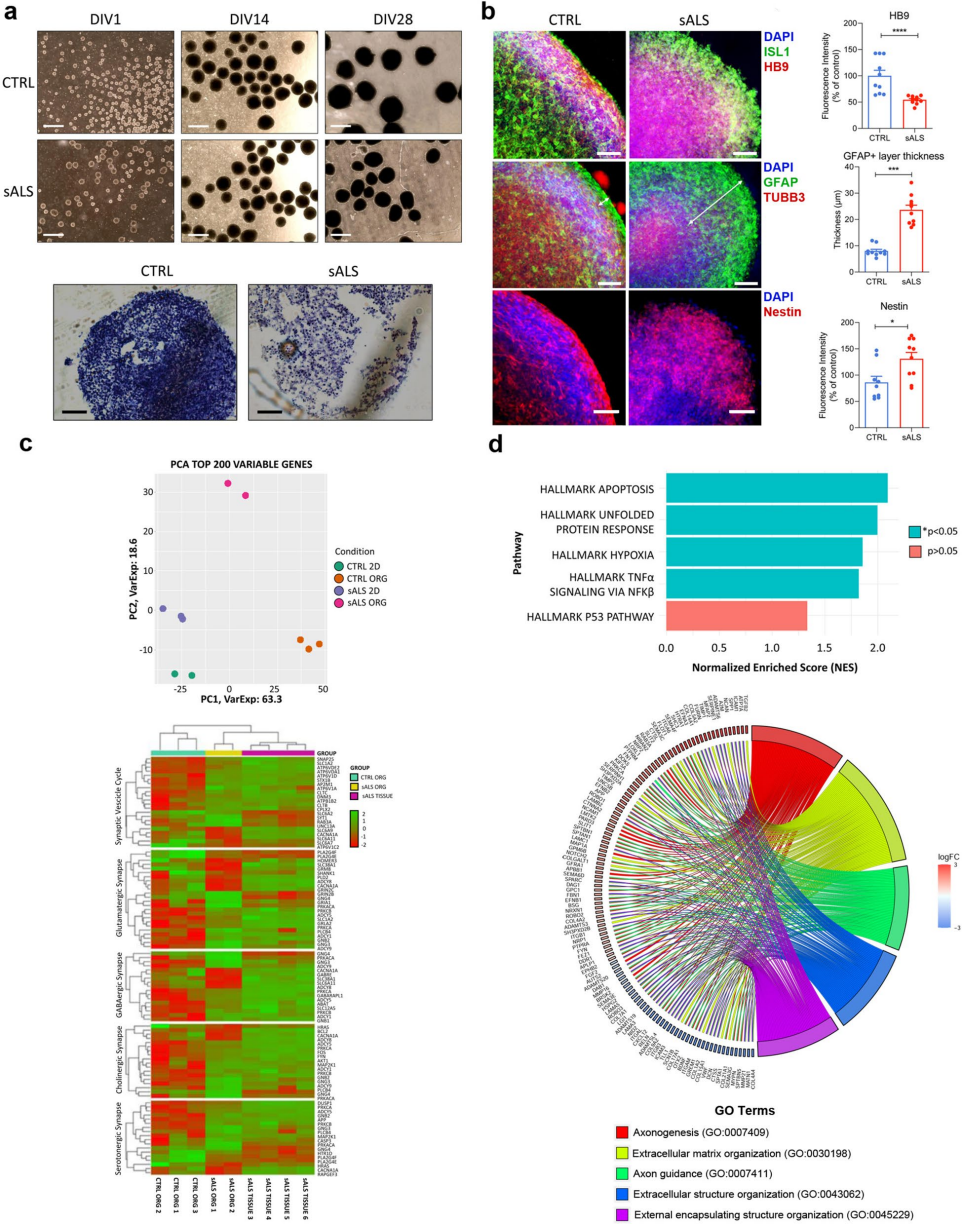
Morphological and transcriptional alterations in sALS SCOs. **a** Representative light field microscopy images at DIVs 1, 14 and 28 of both CTRL and sALS SCOs; scale bar = 500 µm. Morphological analysis by Nissl staining at 20X magnification. **b** Representative images of mature MNs (ISL1 and HB9), glial (GFAP) and neural (TUBB3) cells, and neural progenitor’s cells (Nestin) analyzed via confocal microscopy; scale bar: 50 μm. IF analysis revealed a significant decrease (****p < 0.0001) in the number of HB9 + cells in sALS SCOs, a significant increase in the thickness of the layer of GFAP + cells (white double arrows) (****p < 0.0001), a significant increase (*p = 0.0133) in Nestin + cells in sALS SCOs; N (number of SCOs analyzed for each sample) = 10. **c** PCA of the 200 top DE genes. PCA was performed between CTRL 2D MNs (green), CTRL SCOs (orange), sALS 2D MNs (violet) and sALS SCOs (pink). PCA revealed greater separation between the CTRL and sALS SCOs than between the CTRL and sALS 2D MNs. Moreover, the transcriptional profiles of sALS SCOs were comparable to those of native tissue in terms of synaptic processes highlighted by KEGG analysis. **d** GSEA revealed a statistically significant positive enrichment (NES > 1) for the apoptosis, unfolded protein response, hypoxia, and TNFα signaling via NF-κB pathways. GO chord analysis of SCOs revealed that axonogenesis, ECM organization, axon guidance, extracellular structure and external encapsulating structure organization morphogenesis were the 5 top DE biological processes. All the data were analyzed using t-test followed by the Mann–Whitney test (GraphPad Prism 9)

Moreover, to investigate the molecular profile of the SCO model, RNA-seq analysis was performed to compare transcriptome profiles between classical two-dimensional (2D) MN cultures (sALS vs CTRL) and SCOs (sALS vs CTRL) derived from the same subjects. These lines were previously characterized in another study [4]. In 2D MNs, a total of 382 differentially expressed (DE) genes were identified (71 downregulated and 311 upregulated), whereas SCOs exhibited substantially more transcriptional changes, with 2,929 DE genes detected (1,322 downregulated and 1,607 upregulated). Principal component analysis (PCA) of the top 200 DE genes revealed a clear separation among all groups when analyzed together (Fig. 1c). Notably, the separation observed in SCOs was much more pronounced than that in 2D MNs (Fig. 1c). To assess whether SCOs could serve as a valid experimental model of the human spinal cord, we evaluated the correlation between sALS SCOs and native spinal cord tissue. This analysis showed that sALS SCOs exhibit a transcriptional DE profile comparable to that of sALS spinal cord tissue, indicating that SCOs capture substantially greater biological complexity (Fig. 1c). Consistent with our previous observations, gene set enrichment analysis (GSEA) revealed significant enrichment of multiple pathways associated with cellular stress responses and programmed cell death (Fig. 1d). A normalized enrichment score (NES) > 1 indicates that genes in these pathways are predominantly enriched at the top of the ranked gene list, reflecting an overall upregulation of the pathway-associated transcriptional program. These data support a transcriptional landscape consistent with increased cellular stress and pro-death signaling, reinforcing our hypothesis of enhanced neuronal loss. Moreover, gene ontology (GO) term enrichment analysis of DE genes in sALS SCOs identified the top five deregulated pathways: axonogenesis, extracellular matrix (ECM) organization, axon guidance, extracellular structure, and external encapsulating structure organization (Fig. 1d). The deregulation of the nine most significant ECM-related genes was validated by qRT‒PCR (*p < 0.05: OPN, NCAN, TIMP1, FLOT1, CTSL, FN1, EFNB3, COL4A4; ****p < 0.0001: TIMP2). These findings support the hypothesis that ECM impairment is a pathogenic feature in sALS. Indeed, ALS etiopathogenesis has been linked to ECM dysregulation, which can impair normal intercellular communication [5]. SCOs provide a platform to study ECM pathways in detail, including OPN, which has emerged as a potential biomarker of ALS [5].

In this study, we demonstrated that the reported SCOs recapitulate many pathological features of sALS. This work provides a proof-of-concept for a new protocol to generate human SCOs, although our findings should be confirmed in a larger cohort. We performed three replicates using one CTRL and one sALS subject; therefore, additional samples are required to strengthen the model, as our data may reflect inter-individual variability and should be interpreted with caution. Furthermore, the study lacks functional validation, including calcium imaging and multi-electrode array recordings, and does not include apoptosis or cell death assays, which would be necessary to directly assess neuronal degeneration. Finally, bulk RNA-seq was used in this study; future investigations using single-cell RNA-seq will be essential to examine cell type-specific changes in greater detail.

## Supplementary Information

Supplementary Material 1.

## Data Availability

The raw data obtained from the RNA-seq analysis are deposited in the Gene Expression Omnibus repository [accession number GSE217876].
