# Supplementary material for: Development and characterization of 3D spinal cord organoids to advance the study of amyotrophic lateral sclerosis
Source: Mol Biomed. 2026 Apr 24;7:57. doi: 10.1186/s43556-026-00453-0 (PMC13109475; doi:10.1186/s43556-026-00453-0)
Supplement: Supplementary file 1 — Supplementary Material 1. [file 43556_2026_453_MOESM1_ESM.docx]

**Title: Development and characterization of 3D spinal cord organoids to advance the study of amyotrophic lateral sclerosis**

Matteo Bordoni^1^, Eveljn Scarian^1^, Letizia Messa^2,3^, Maria Garofalo^4^, Emanuela Jacchetti^5^, Manuela Teresa Raimondi^5^, Luca Diamanti^6^, Stella Gagliardi^4^, Stephana Carelli^3,7#^, Cristina Cereda^3,7#^, Orietta Pansarasa^1#*^

1) Cellular Models and Neuroepigenetics Unit, IRCCS Mondino Foundation, 27100 Pavia, Italy; MB: matteo.bordoni@mondino.it, ES: eveljn.scarian@mondino.it, OP: orietta.pansarasa@mondino.it

2) Department of Electronics, Information and Bioengineering (DEIB), Politecnico di Milano, 20133 Milano, Italy; LM: letizia.messa@polimi.it

3) Center of Functional Genomics and Rare Diseases, Department of Pediatrics, Buzzi Children's Hospital, 20154 Milano, Italy; CC: cristina.cereda@asst-fbf-sacco.it

4) Molecular Biology and Transcriptomics Unit, IRCCS Mondino Foundation, 27100 Pavia, Italy; MG: garofalomaria.bio@gmail.com, SG: stella.gagliardi@mondino.it

5) Department of Chemistry, Materials and Chemical Engineering “Giulio Natta”, Politecnico di Milano, 20133 Milano, Italy; EJ: emanuela.jacchetti@polimi.it, MTR: manuela.raimondi@polimi.it

6) Neuroncology Unit, IRCCS Mondino Foundation, 27100 Pavia, Italy; LD: luca.diamanti@mondino.it

7) Pediatric Clinical Research Center "Romeo ed Enrica Invernizzi", Department of Biomedical and Clinical Sciences, University of Milano, 20157 Milano, Italy; SC: stephana.carelli@asst-fbf-sacco.it

#These authors share last authorship

*Corresponding author:

Orietta Pansarasa

e-mail: orietta.pansarasa@mondino.it

**Methods**

*Differentiation of iPSC into 2D MNs*: One iPSC line derived from a non-mutated sALS patient (male, 57 years old) and one from a healthy donor (male, 59 years old) were used. The aim was to reduce inter-individual variability in order to assess the robustness of the organoid model and the intrinsic differences between 2D and 3D culture systems. All experiments were performed in triplicate from independent differentiations. Briefly, iPSCs were differentiated into neural stem cells (NSCs) using a medium composed of Neurobasal (21103049) and Neural Induction Supplement 50X (A1647801) for 7 days and then expanded in a medium composed of Neurobasal, Advanced DMEM/F12 (12634010) and Neural Induction Supplement 50X. NSCs were differentiated into motor neuron progenitors (MNPs) using a medium composed of Neurobasal, Advanced DMEM/F12, Neural induction supplement 50X, 0.1 µM Retinoic Acid (R2625, Merck) and 0.5 µM Purmorphamine (540220, Merck). After 7 days MNPs formed and were induced to differentiate into MNs in a two step-protocol: cells were first cultured for 7 days in a medium composed of Neurobasal, Advanced DMEM/F12, 0.5 µM Retinoic Acid, 0.1 µM Purmorphamine, 10 ng/mL glia derived neurotrophic factor (PHC7045), 10ng/mL insulin growth factor (PHG0071) and 10 ng/mL brain derived neurotrophic factor (PHC7074) for the formation of immature MNs; after 7 days, 0.1 µM Compound E (209986-17-4, Santa Cruz Biotechnology) was added to the medium for the complete maturation of MNs. All reagents were purchased from ThermoFisher unless otherwise specified.

*Generation of SCOs*: NSCs were split using StemPro Accutase (A1110501, ThermoFisher) and filtered through a 40 µm cell strainer for complete dissociation. For each sample, 4 × 10^6^ NSCs were then seeded on a non-coated 6-well plate and cultured under floating conditions on an Orbi-Shaker CO2 (BT4001, Benchmark Scientific) at 95 rpm. NSCs were then differentiated in suspension with the same media described in the previous section.

*Phase-contrast microscopy analysis*: SCOs morphology was evaluated by phase-contrast microscope EVOS™ XL Core Imaging System (AMEX1000, ThermoFisher) during all the differentiation stages.

*Nissl staining*: SCOs at DIV28 were fixed in 4% paraformaldehyde (1004968350, Merck) at 4°C overnight (ON). SCOs were then incubated in 20% sucrose (8590-OP, Merck) solution and transferred to a cryosection mold. SCOs were then incubated for in a 1:1 mixture of 30% sucrose (8590-OP, Merck) and optimal cutting temperature (OCT) compound (4583, Sakura Finetek) and frozen in liquid nitrogen vapor. The samples were cryosectioned into 12 μm slices using LEICA CM1520 cryostat (LEICA Biosystems). Nissl staining was performed by hydration steps, incubation with 0.1% cresyl violet solution (ab246816, Abcam) for 10 min, and finally dehydration steps.

*Immunofluorescence analysis*: SCOs at DIV28 were fixed in 4% paraformaldehyde (1004968350, Merck) and then blocked in 5% normal goat serum (31872, ThermoFisher) and 0.1 % Tween-20 (P9416, Merck) in 1X PBS (P4417, Merck). SCOs were incubated with primary antibodies at 4°C ON, and the next day they were incubated with secondary antibodies. After an attachment phase on polylysine slides (P0425, Merck), SCOs were mounted with the Prolong® Gold antifade reagent DAPI (P36935, ThermoFisher), dried and nail-polished. Images were acquired by using confocal microscope (Nikon-A1 Confocal Microscope). IF analysis was performed with ImageJ software.

*Bulk RNA sequencing*: Libraries were prepared starting from 500 ng of total RNA. Three libraries were generated from independent experiments for each sample. The RNA was fractioned for rRNA depletion using RiboCop V1.3 (037, Lexogen GmbH), and the libraries were prepared with the CORALL Total RNA-Seq Library Prep Kit (096, Lexogen). The quality of the libraries was analyzed using 4200 Tape Station (G2991BA, Agilent) with a DNA High Sensitivity assay (5067-4626, Agilent) and quantified using High Sensitivity dsDNA assay (Q32851, ThermoFisher) with a Qubit device (Q33216, ThermoFisher). Sequencing was performed using Illumina NextSeq 500 (SY-415-1001, Illumina), and FASTQ files were generated via an Illumina bcl2fastq2, version 2.17.1.14 (http://support.illumina.com/downloads/bcl-2fastq-conversion-software-v217.html), starting from raw sequencing reads produced by Illumina NextSeq sequencer.

*Bioinformatic analysis*: FASTQ generated by Illumina NextSeq 500 (SY-415-1001, Illumina) sequencer through Unique Molecular Identifiers (UMI) extraction, trimming, alignment and quality control steps. As CORALL libraries contain N12 UMI at the start of Read 1, in the first step, UMI were removed through UMI tools software. Then, the adapter sequences were trimmed through Cutadapt software. After UMI extraction and trimming, trimmed reads were aligned through STAR using Gencode Release h38 (GRCh38). Gene and transcript abundance were computed using FeatureCounts software. Differential expression analysis was performed using R package DESeq2. The design matrix was built considering only the different conditions (i.e., CTRL 2D MNs, sALS 2D MNs, CTRL SCOs and sALS SCOs) since no experimental batch effects or other relevant covariates were present. The significance of differential expression between conditions was determined through a likelihood ratio test, which compares the full model, with the variables of interest, to the reduced model, without those variables. The resulting p values were adjusted for multiple testing using Benjamini-Hochberg correction to control the FDR. Genes were considered differentially expressed and retained for further analysis with |log2(condition sample/control sample) | ≥ 1 and an FDR ≤ 0.1. R software was used to generate heatmaps (heatmap.2 function from the R ggplots package), PCA plots of the top 200 most DE genes (prcomp function from the R ggplots package), GSEA (fgsea package) and GO Chord plot (GOChord function from the R GOplot package). Functional enrichment analysis was performed on the differentially expressed coding genes via the enrichR web tool. Finally, the raw FASTQ files from the ALS patient spinal cord tissue dataset (SRP064478) were processed using the same bioinformatics pipeline applied to SCOs.

*Statistical analysis*: The generation and cultivation of SCOs were performed at three different times for each condition in a completely independent way. For each assay, at least three independent experiments were performed to establish the reproducibility of the measurements. Statistical analyses were performed using GraphPad Prism 9, adopting unpaired t-test followed by the Mann–Whitney test. For evaluating the differences in size considering both condition and time, we used the two-way ANOVA test. The data are reported as the means ± standard deviations. No blinding was performed.
